# Microinflammation-Driven Gene Expression Dynamics in the Pathogenesis of Metabolic Disorders and Cancer

**DOI:** 10.3390/biology15010019

**Published:** 2025-12-21

**Authors:** Marian Elisa Gabrielle T. Cadungog, Lemmuel L. Tayo

**Affiliations:** 1 School of Chemical, Biological, and Materials Engineering and Sciences, Mapúa University, Manila 1002, Philippines; megtcadungog@mymail.mapua.edu.ph; 2 School of Graduate Studies, Mapúa University, Manila 1002, Philippines; 3 Department of Biology, School of Health Sciences, Mapúa University, Makati 1203, Philippines

**Keywords:** microinflammation, RNA processing, spliceosome, ribosome biogenesis, proteostasis, microRNAs, metabolic reprogramming, inflammation–metabolism crosstalk, cancer–metabolic disease interaction

## Abstract

Microinflammation serves as a central mechanism linking metabolic diseases and cancer. This study integrates gene expression profiles from irritable bowel syndrome (IBS), obesity, type 2 diabetes (T2D), colorectal cancer (CRC), renal cell carcinoma (RCC), and pancreatic cancer (PC) to identify shared molecular drivers of inflammation-mediated pathology. Weighted gene co-expression network analysis (WGCNA) revealed three highly preserved modules (blue, brown, turquoise) enriched in RNA processing, spliceosome assembly, ribosome biogenesis, and proteostasis regulation. Key hub genes, along with regulatory miRNAs have interconnected networks that modulate transcription, mRNA maturation, protein synthesis, and inflammatory signaling. Although classical inflammatory pathways were not directly enriched, their activity appears to be indirectly shaped by disruptions in RNA-processing and proteostasis machinery. Additionally, gut microbiota-derived products and altered metabolic states may further reinforce these transcriptional and post-transcriptional imbalances. Collectively, these findings reveal conserved molecular signatures that bridge microinflammation, metabolic disease, and oncogenesis, and highlight potential diagnostic and therapeutic targets centered on RNA regulation, proteostasis, and miRNA-mediated control

## Simple Summary

This study explores how microinflammation—a subtle, long-lasting form of inflammation—connects common metabolic conditions like obesity, type 2 diabetes, and irritable bowel syndrome with cancers of the colon, kidney, and pancreas. By analyzing gene activity across multiple datasets, the research found shared disruptions in how cells process genetic information and manage proteins. Key genes and small regulatory molecules appear to drive these changes and sustain chronic inflammation. These findings suggest that many chronic diseases are linked by the same underlying biological processes. Understanding these connections may support earlier diagnosis and lead to treatments that target shared molecular pathways, offering broad benefits for preventing or slowing both metabolic disorders and cancer.

## 1. Introduction

Microinflammation is a subtle, persistent form of inflammation that occurs at a microscopic (cellular or tissue) level, often without obvious clinical symptoms. Unlike acute inflammation, which is typically short-lived and easily detectable, microinflammation is low-grade and chronic, greatly driven by factors such as oxidative stress, metabolic imbalance, nutritional deficiencies, and immune dysregulation [1]. In metabolic disorders like obesity and type 2 diabetes, microinflammation alters gene expression and disrupts cellular homeostasis, promoting insulin resistance and organ dysfunction. Similarly, in cancer, it can create a pro-tumorigenic microenvironment that supports malignant transformation and progression [2]. Moreover, microinflammation is now increasingly correlated in cardiovascular disease, neurodegenerative conditions, and autoimmune disorders, highlighting its central role in chronic disease development and the need for deeper investigation into its molecular mechanisms and therapeutic targeting [3].

Metabolic diseases have been on the rise worldwide, placing an increasing burden on public health infrastructure. Most individuals encounter microinflammation in everyday life—from stress, poor diet, sleep disruption, or environmental exposure—without realizing that such subtle immune activation can predispose them to chronic disorders. This silent inflammatory state of affected individuals has emerged as a critical contributor to the pathogenesis of various chronic diseases Irritable bowel syndrome (IBS), for instance, affects 10–15% of the global population and causes diminished quality of life and excessive healthcare utilization Type 2 diabetes (T2D) and obesity have reached epidemic proportions, with more than 500 million individuals currently living with T2D [4]. These conditions share overlapping biological mechanisms, including chronic low-grade inflammation, impaired metabolism, and gut microbiota imbalance. Obesity and T2D are confirmed risk factors for multiple cancers, including colorectal cancer (CRC), renal cell carcinoma (RCC), and pancreatic cancer (PC). These diseases altogether account for a significant proportion of cancer-related deaths worldwide [5,6].

IBS, a functional gastrointestinal disorder marked by abdominal pain, bloating, and irregular bowel movement, exemplifies how persistent low-grade inflammation silently shapes disease risk. It is increasingly associated with intestinal immune activation, microbiota dysbiosis, and gut–brain axis disruption [7,8]. Similarly, obesity and T2D, often co-manifesting as metabolic syndrome, are characterized by insulin resistance, dyslipidemia, and systemic inflammation that drive oxidative stress and immune dysregulation [9,10]. These effects extend to the gut, fostering a pro-tumorigenic microenvironment. Among malignancies, CRC ranks as the third most common cancer and a leading cause of cancer-specific deaths globally [11]. RCC accounts for around 3% of adult malignancies, with clear cell RCC (ccRCC) as the predominant subtype [12], while pancreatic cancer—though relatively rare—remains one of the deadliest cancers, with a five-year survival rate below 10% [13]. While many other conditions—such as liver cirrhosis, autoimmune disorders, and chronic infections (e.g., HSV, TB, HCV, HIV)—are also characterized by persistent microinflammation, they were not included in this study because comparable, high-quality transcriptomic datasets suitable for harmonized integration were not available across these diseases. Therefore, we focused on IBS, T2D, obesity, and major gastrointestinal cancers, which share overlapping inflammatory and metabolic features and for which sufficiently consistent datasets exist. These additional conditions remain important contexts in which similar mechanisms may operate and represent valuable directions for future investigation.

The crosstalk between genetic factors, lifestyle determinants, and persistent inflammation likely underlies the development of these diseases. Emerging research points to shared mechanisms among IBS, obesity, T2D, and cancer, with chronic inflammation as a unifying theme: IBS involves mucosal immune activation, while obesity and T2D are marked by elevated cytokines such as TNF-α and IL-6 [14,15,16]. These inflammatory mediators drive DNA damage, angiogenesis, and cell proliferation, fueling tumor initiation and progression. Gut microbial dysbiosis further links these disorders—altered microbial composition and metabolites compromise epithelial integrity and modulate the immune microenvironment, promoting carcinogenesis [17]. Metabolic dysregulation amplifies this by enhancing proliferation and blocking apoptosis [18], while genetic and epigenetic changes in inflammatory mediators and tumor-suppressor pathways heighten susceptibility to cancer [19].

Despite their interconnected biology, current therapies treat these conditions in isolation and often yield suboptimal outcomes. IBS management remains largely symptomatic, relying on diet and neuromodulators [20]; obesity and T2D therapies center on lifestyle and pharmacologic interventions, with adherence challenges and complications persisting [21]; and although targeted and immune therapies have extended survival in cancer, recurrence and drug resistance remain major obstacles [22]. These gaps underscore the need to investigate shared molecular mechanisms rather than isolated pathologies.

Another critical regulatory layer involves microRNAs (miRNAs), post-transcriptional regulators that bind messenger RNAs (mRNAs) to control their stability and translation. Because each miRNA can modulate multiple targets simultaneously, they fine-tune entire pathways and amplify or suppress cellular responses [23,24]. Dysregulated miRNAs contribute to inflammation, metabolic disruption, immune evasion, and tumor progression across metabolic diseases and cancers [25,26,27]. Notably, miR-21, miR-155, and miR-34a govern pathways such as PI3K/AKT, NF-κB, and p53 that regulate proliferation, apoptosis, and metabolic control [28,29]. This multitarget regulatory capacity positions miRNAs as master switches where metabolic dysregulation and oncogenic signaling intersect. Mapping miRNA–gene interactions within preserved co-expression modules thus offers mechanistic insight into how gene networks co-regulate IBS, obesity, T2D, and cancer. Integrating miRNA data with hub genes can reveal early biomarkers or therapeutic targets capable of restoring molecular balance across multiple diseases [30].

This study utilized six gene expression datasets (GSE152991, GSE164416, GSE146853, GSE213324, GSE50760, and GSE211398) to perform an integrated bioinformatics analysis. Weighted gene co-expression network analysis (WGCNA), functional enrichment tools, and protein–protein interaction (PPI) networks were used to identify shared modules and hub genes among IBS, T2D, obesity, CRC, RCC, and pancreatic cancer. Despite representing distinct clinical conditions, IBS, T2D, obesity, colorectal cancer, renal cell carcinoma, and pancreatic cancer share several convergent biological features, including chronic low-grade inflammation, metabolic reprogramming, epithelial barrier dysfunction, and altered RNA and protein homeostasis. These overlapping hallmarks suggest that common upstream regulatory mechanisms may operate across metabolic, inflammatory, and tumorigenic states. Integrating transcriptomic datasets from these diseases enables the identification of conserved co-expression modules and regulatory pathways that transcend disease boundaries, providing a systems-level view of shared molecular architecture. To ensure that differences across datasets did not introduce technical bias, all studies were processed using an identical normalization pipeline (quantile normalization, log_2_ transformation, background correction, and removal of control probes), and only genes present in all datasets were retained for analysis. This harmonized preprocessing approach allows for robust cross-disease integration while minimizing platform or study-specific variability. Experimentally validated and predicted miRNA–mRNA interactions were then integrated with the hub genes to construct regulatory networks, facilitating the identification of potential miRNA–hub gene axes that may serve as diagnostic or therapeutic biomarkers.

## 2. Materials and Methods

### 2.1. Data Retrieval and Preprocessing

Expression data of the RNA analyses were obtained from the National Center for Biotechnology Information of the Gene Expression Omnibus (NCBI) [https://www.ncbi.nlm.nih.gov/geo/, accessed on 3 June 2025]. We analyzed 190 samples: 34 of obesity, 37 of Type 2 Diabetes (T2D), 46 of irritable bowel syndrome (IBS), 21 of renal cell carcinoma (RCC), 36 of Colorectal Cancer, and 16 of Pancreatic Cancer. Details and distribution of the samples on the various diseases were presented in Table 1. The datasets include healthy controls as well as cases of IBS, T2D, obesity, and RCC, colorectal, and pancreatic tumors, but only the disease-related samples were selected for analysis.

All raw data were examined in R v(.4.4.0) through the deseq2 package of Bioconductor (www.bioconductor.org, accessed on 3 June 2025). Quantile normalization, log-2 transformation, and background correction were performed on all datasets. Boxplots and sample clustering dendrograms were applied to detect outliers. During preprocessing, the control probes were excluded to suppress non-biological variation, and the gene expression data were filtered with the inclusion of those having mean and variance greater than the 20th percentile. AnnotationDbi function and the hgu133plus2.db database were used to change the probe IDs to gene symbols to facilitate downstream biological interpretation. Probes that were common to all the datasets were maintained, and samples with zero values after log-2 transformation were excluded by the goodSamplesGenesMs function of the WGCNA R package [31,32].

### 2.2. Weighted Gene Co-Expression Network Analysis (WGCNA)

#### 2.2.1. Scale-Free Network Approximation

The appropriate soft-thresholding power (β) was determined by the WGCNA R package pickSoftThreshold function by graphing scale-free topology fit against power indices (1–20) and taking the lowest power that satisfied the scale-free topology requirement. In scale-free topology, gene connectivity follows a power-law distribution that is assessed by looking for linear correlation between the log of connectivity and the log of the connectivity probability. To select the appropriate soft-thresholding power (β), the scale-free topology fit was graphed against different estimates of powers, selecting the lowest β where the fit stabilized or reached threshold level. The chosen β was also validated by generating approximate straight-line relations by the soft-connectivity (k) values of each dataset, along with ranked expression and ranked soft-connectivity graphs to validate dataset comparability before network construction [33,34].

#### 2.2.2. Network Construction and Module Identification

For building the network, Pearson correlation produced the adjacency matrices with the ‘signed’ network type, and the topological overlap measure (TOM) dissimilarities were calculated by raising the adjacency matrices to the desired soft-thresholding power (β). This favors strong correlations and downweights weak correlations, permitting effective hierarchical gene clustering by the flashClust function. The gene dendrograms were then produced by the hclust function with the ‘average’ method. Modules were identified using hierarchical clustering with the cutreeHybrid function, testing deep split parameters ranging from 0 to 3 to assess branch-splitting sensitivity [35,36]. Robust modules amidst variable parameter settings showed robust clustering, but variable patterns showed the need for further parameter validation, such as β.

### 2.3. Module Preservation Analysis

Module preservation analysis was performed to find out if the gene co-expression patterns of the modules are conserved across different datasets and conditions and therefore deduce their biological significance. It was conducted using the modulePreservation function of the WGCNA R package with a ‘signed’ type of network, 1000 permutations, and a minimum of 100 module size. The eigengene-based connectivity (kME), i.e., the correlation measure of the single genes with their module eigengenes, was calculated by the moduleEigengenes function to identify core module genes and deduce the module membership [37].

### 2.4. Gene Ontology (GO) and Kyoto Encyclopedia of Genes and Genomes (KEGG) Pathways Analysis

Genes of every one of the conserved modules were put through functional and pathway enrichment by the Database for Annotation, Visualization, and Integrated Discovery (DAVID) (https://davidbioinformatics.nih.gov/, accessed on 3 June 2025). Gene Ontology (GO) evaluation included biological processes (BP), molecular functions (MF), and cellular components (CC), as well as Kyoto Encyclopedia of Genes and Genomes (KEGG) pathway enrichment. Stringency of the classification of the DAVID was medium, and only the significant terms (adjusted *p*-value < 0.05) with enrichment scores greater than 1.3 were considered. Significant KEGG pathways that were clustered with enriched GO terms were also analyzed further to detect the biological functions of the conserved modules [38,39].

### 2.5. Protein–Protein Interaction (PPI) Network Analysis and Hub Genes

Protein–protein interaction (PPI) networks of each conserved module were generated with STRING v12.0 (https://string-db.org/, accessed on 10 June 2025) by employing a high-confidence interaction score cutoff of 0.7. The generated networks were imported into Cytoscape v3.10.1 for the purpose of topological analysis, where hub genes were identified by making use of the CytoHubba (Cytoscape v3.10.) plugin. Several centrality measures like degree, closeness, betweenness, and maximum neighborhood component (MNC) were put into use, and the top-scoring genes from each of these were made comparable to one another. Genes that were regularly picked by the different metrics were deemed hub genes, and a distinct PPI network was generated to display their interactions [40].

### 2.6. Hub miRNA Identification

The identified hub genes were uploaded into the miRNet platform (https://www.mirnet.ca/, accessed on 3 June 2025), a miRNA-centric network visual analytics tool. miRNet integrates functional information from 14 curated databases, including miRTarBase, which provide experimentally validated and predicted miRNA–gene interactions. Only inetractions validated by strong evidence, i.e., Reporter Assay, Western blot, and qPCR were selected. A gene–miRNA interaction network was generated, where nodes represented hub genes and miRNAs, and edges indicated their regulatory associations. The network was constructed and visualized using the built-in force-directed layout algorithms provided by miRNet. To ensure data reliability, unknown entries were excluded from the network. From the remaining nodes, only the top 10 miRNAs were retained and selected based on degree centrality, retaining those with the highest number of connections to hub genes as the most influential regulators for downstream analysis.

## 3. Results

### 3.1. Weighted Gene Co-Expression Network Analysis (WGCNA)

#### 3.1.1. Scale-Free Network Analysis of the Datasets

**Figure 1:**
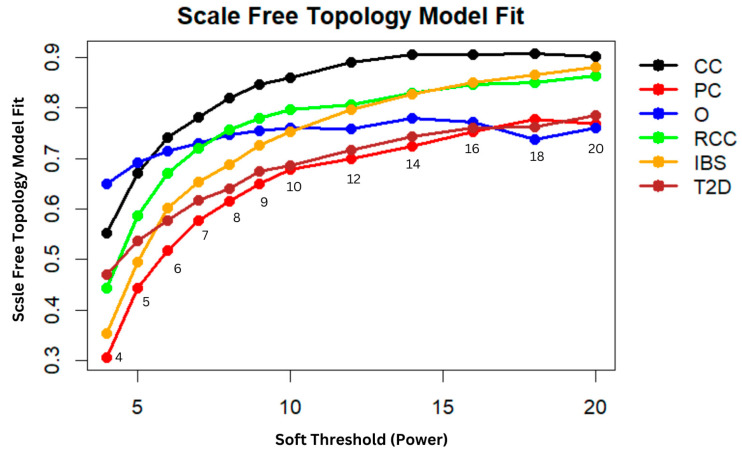
Network indices were used to approximate a scale-free topology by evaluating the average number of connections per gene across the networks.

Weighted gene co-expression network analysis (WGCNA) has the gene expression matrices first normalized and the outliers filtered out by screening to be left with high quality information to be analyzed. After the filtering process, the overlapping number of the dataset’s genes were utilized to construct the networks that boost the robustness of the analysis due to the large number of the genes that were considered. The soft-thresholding power (β) was therefore determined by searching the scale-free topology fit index versus different β powers (β = 1–20). As shown in Figure 1, the fit index converged at β = 10, indicating that larger β powers could not improve the scale-free topology of the network further. Therefore, β = 10 was selected for all the datasets, which produced the optimal balance between biological relevance and network sparseness. No sample outliers that were significantly different from the others were revealed by the clustering dendrograms, and therefore all the samples were considered in the study. Taking β = 10 ensures the constructed gene networks to be robust, efficient, and meaningful biologically. Selecting the smallest β at which the topology fit index is stabilized suppresses the noise and preserves true gene–gene interactions, yielding modules that more accurately capture underlying biological phenomena.

**Figure 2:**
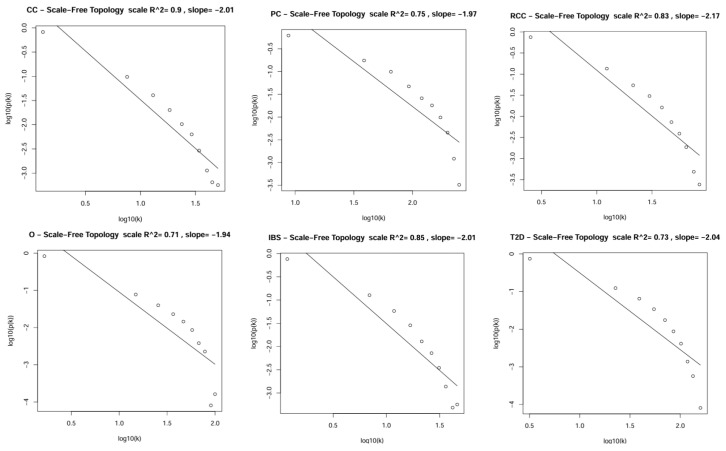
The log–log plot for the datasets illustrates an approximately linear relationship between node degree (k) on the X-axis and its probability p(k) on the Y-axis at β = 10, confirming the scale-free topology of the network.

Figure 2 shows that the highest scale-free topology correlation was reached by the datasets, which had varying R^2^. This high correlation reflects that the dataset is suitable for the construction of WGCNA networks and the identification of modules as it contains biologically meaningful patterns of expression [30]. Since R^2^ of around 0.9 is generally believed to be the optimum, the high value also reflects the belief that biological networks have a scale-free topology that further supports the suitability of the dataset to discern functionally relevant modules of genes.

#### 3.1.2. Scale-Free Network Approximation

**Figure 3:**
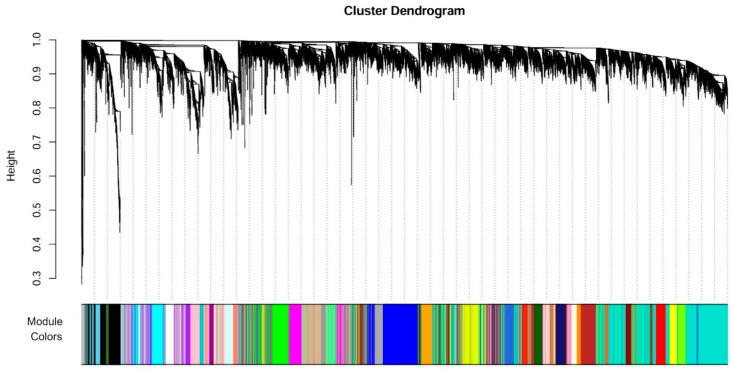
Dendrogram of gene clustering and module identification for the datasets.

In WGCNA, meta-analysis comprises mapping module eigengenes of numerous diverse datasets on a select reference dataset, which is essential to yielding network robustness and appropriate clustering by the topological overlap matrix (TOM). For the scope of the current study, datasets were chosen as references due to their very good scale-free topology fitting in addition to robust gene expression patterns. Soft-thresholding of the power β = 10 was utilized to construct the adjacency matrix as well as the dendrogram of the genes. As shown by Figure 3, some distinct gene co-expression modules were discerned by WGCNA. Sensitivity plots also confirmed the modules’ stability, albeit further increased sensitivity, likely due to robust gene correlations or the presence of fewer, more biologically informative, clusters.

Using TOM similarity, WGCNA identified forty-two co-expression modules, each labeled with a distinct color: turquoise (2338 genes), blue (791), brown (647), red (453), light yellow (255), midnight blue (291), light cyan (282), and others. The turquoise, blue, and brown modules were the largest, indicating strong gene-to-gene connectivity and suggesting they may capture core, broadly relevant biological processes. Conversely, smaller modules such as white, cyan, and orange emerged only at lower cutting thresholds, reflecting more specific but less interconnected gene clusters. These results highlight TOM’s ability to preserve dense co-expression patterns while also resolving smaller, specialized modules. Overall, the larger modules represent stable, highly interconnected networks, providing a robust framework for cross-disease comparisons.

### 3.2. Module Preservation Analysis

**Figure 4:**
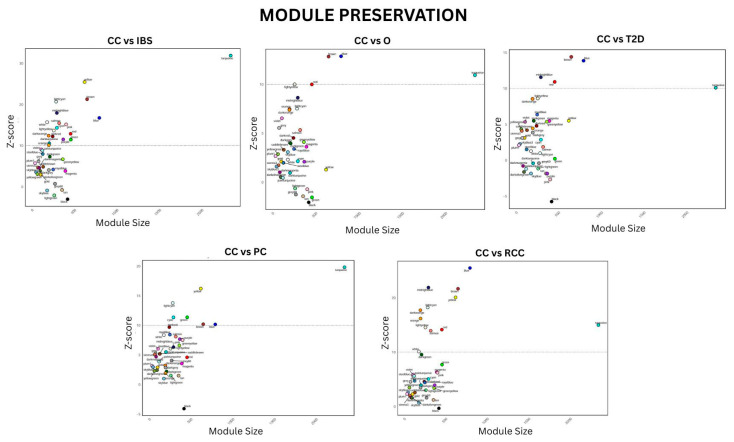
Module preservation analysis performed on the gene co-expression modules from the network.

Module preservation analysis determines the conservation of the identified gene modules between multiple datasets, which indicates the reproducibility and biological relevance of the co-expression networks. Module conservation was high for those modules that had Z-scores greater than 10, and larger modules revealed more significant connectivity patterns. As depicted in Figure 4, most of the modules maintained their relative position across the datasets, and some of these were above the preservation threshold level. The increase in the Z-score values further supports the robustness of the identified modules, revealing that the gene co-expression patterns were uniform across the different conditions.

### 3.3. Gene Ontology (GO) and Kyoto Encyclopedia of Genes and Genomes (KEGG) Pathways Analyses

Eigengene-based connectivity (kME) was calculated by correlation of the gene’s expression profile with the corresponding module eigengene, and the genes were ranked according to kME values. The top-ranking genes of every module were submitted to functional enrichment analysis by DAVID webserver. Gene Ontology (GO) terms—like biological processes (BP), cellular components (CC), and molecular functions (MF)—as well as KEGG pathways were analyzed. As indicated by Figures below, significantly enriched GO terms along with the pathways were given the highest priority when they were concatenated together within the same functional cluster.

#### 3.3.1. Blue Module Result Analysis

**Figure 5:**
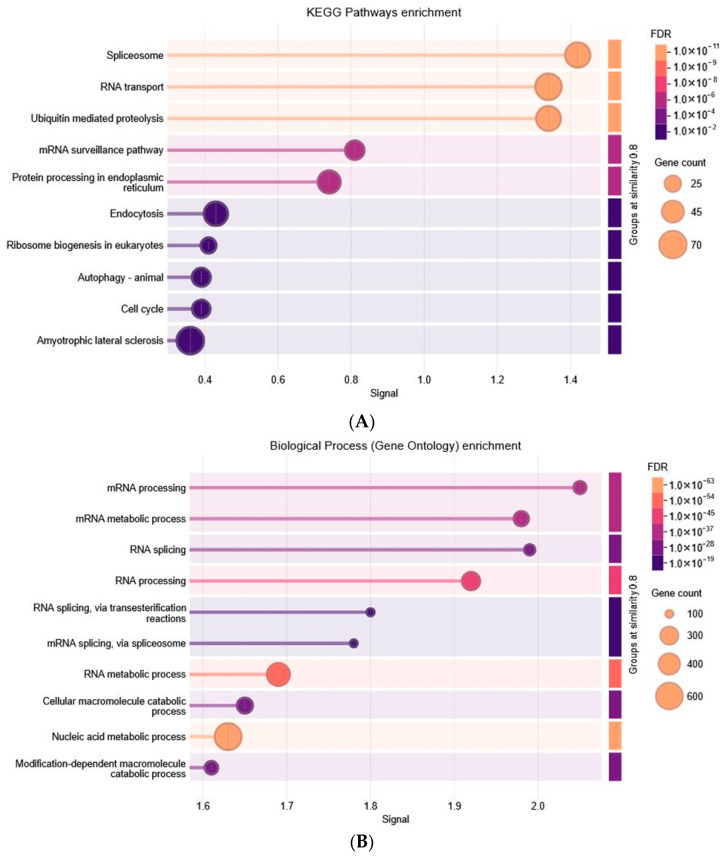

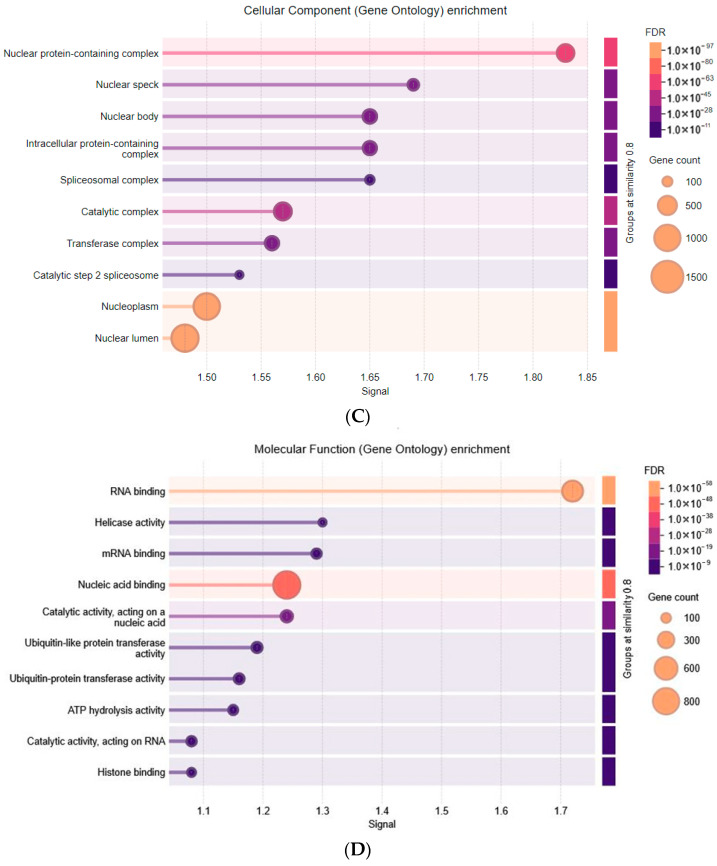
Functional Annotation and Enrichment Analysis of Blue Module. (**A**) KEGG, (**B**) GO BP, (**C**) GO CC, (**D**) GO MF.

The blue module’s hub genes are strongly linked to RNA metabolism, protein balance, and cell-cycle control, which are key processes in cancer and metabolic diseases. Enrichment analysis shows these genes play important roles in both transcriptional and post-transcriptional regulation. KEGG pathway analysis (Figure 5A) highlights mechanisms such as spliceosome, RNA transport, mRNA surveillance, and ribosome biogenesis. The genes are also involved in protein quality control, degradation, and stress responses, which are often disrupted in cancers like colorectal, pancreatic, and renal, as well as in diabetes complications. Further analysis points to enrichment in pathways like ubiquitin-mediated proteolysis, endocytosis, autophagy, cell-cycle regulation, and protein processing in the endoplasmic reticulum. Gene Ontology (GO) analysis (Figure 5B–D) supports these findings, showing enrichment in biological processes such as spliceosome-mediated RNA splicing, nucleic acid metabolism, and mRNA processing. GO cellular component terms cluster these genes in nuclear bodies, spliceosomal complexes, and nuclear specks, which are key sites for transcription and RNA processing. The genes have a wide range of biochemical functions, including RNA binding, helicase activity, mRNA binding, ubiquitin-like protein transferase activity, ATP hydrolysis, and histone binding.

#### 3.3.2. Brown Module Result Analysis

**Figure 6:**
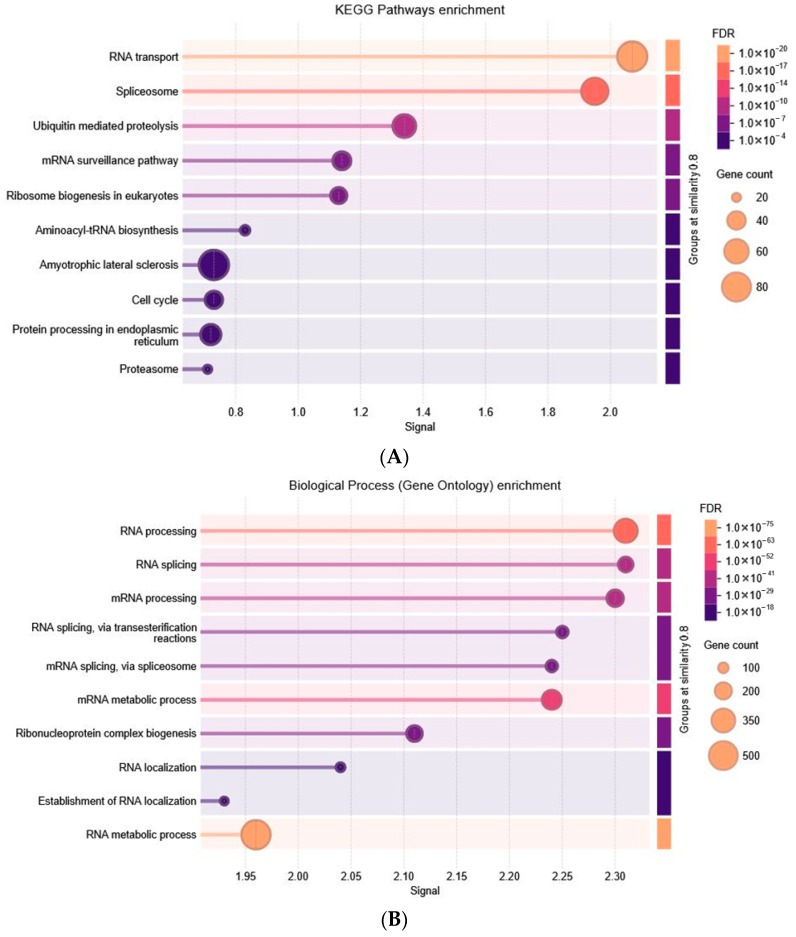

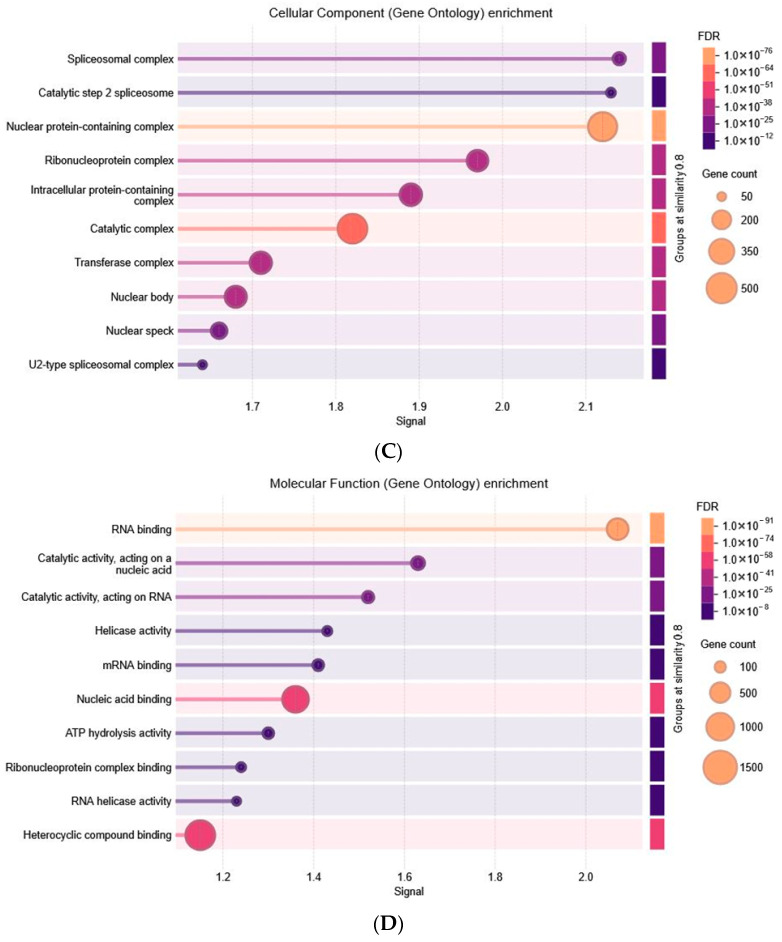
Functional Annotation and Enrichment Analysis of Brown Module. (**A**) KEGG, (**B**) GO BP, (**C**) GO CC, (**D**) GO MF.

Enrichment analysis of the brown module revealed that its hub genes are strongly linked to RNA metabolism and the regulation of splicing. This was evident from significant enrichment in GO biological processes (Figure 6B) such as RNA processing, mRNA splicing via the spliceosome, ribonucleoprotein complex biogenesis, and RNA localization. These findings align with their localization in spliceosomal and ribonucleoprotein complexes, as well as nuclear specks and nuclear bodies, underscoring their central role in RNA maturation and nuclear processing. At the molecular level, the hub genes were enriched for RNA-binding capacity, helicase activity, mRNA binding, ATP hydrolysis, and ribonucleoprotein complex interactions. Such activities are critical for unwinding, stabilizing, and modifying RNA molecules. KEGG pathway analysis (Figure 6A) further supported these results, highlighting enrichment in the spliceosome, mRNA surveillance, RNA transport, ribosome biogenesis, and aminoacyl-tRNA biosynthesis—pathways essential for precise gene expression and protein synthesis. In addition, enrichment in ubiquitin-mediated proteolysis, proteasome activity, protein processing in the endoplasmic reticulum, cell-cycle regulation, and autophagy suggest broader roles in protein quality control, degradation, and cellular stress response.

#### 3.3.3. Turquoise Module Result Analysis

**Figure 7:**
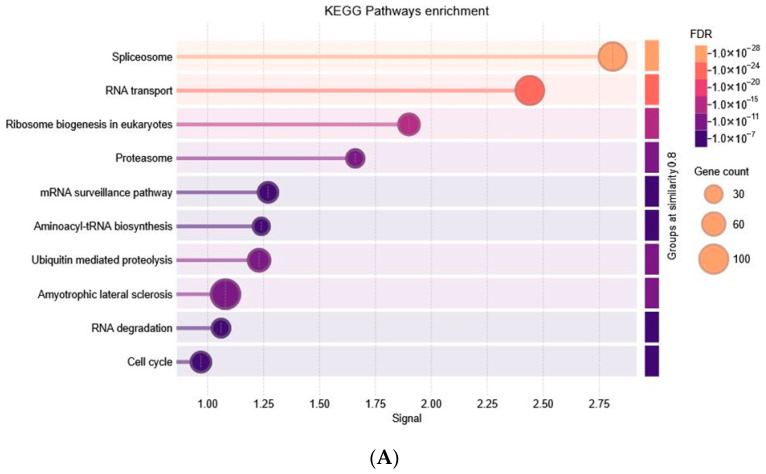

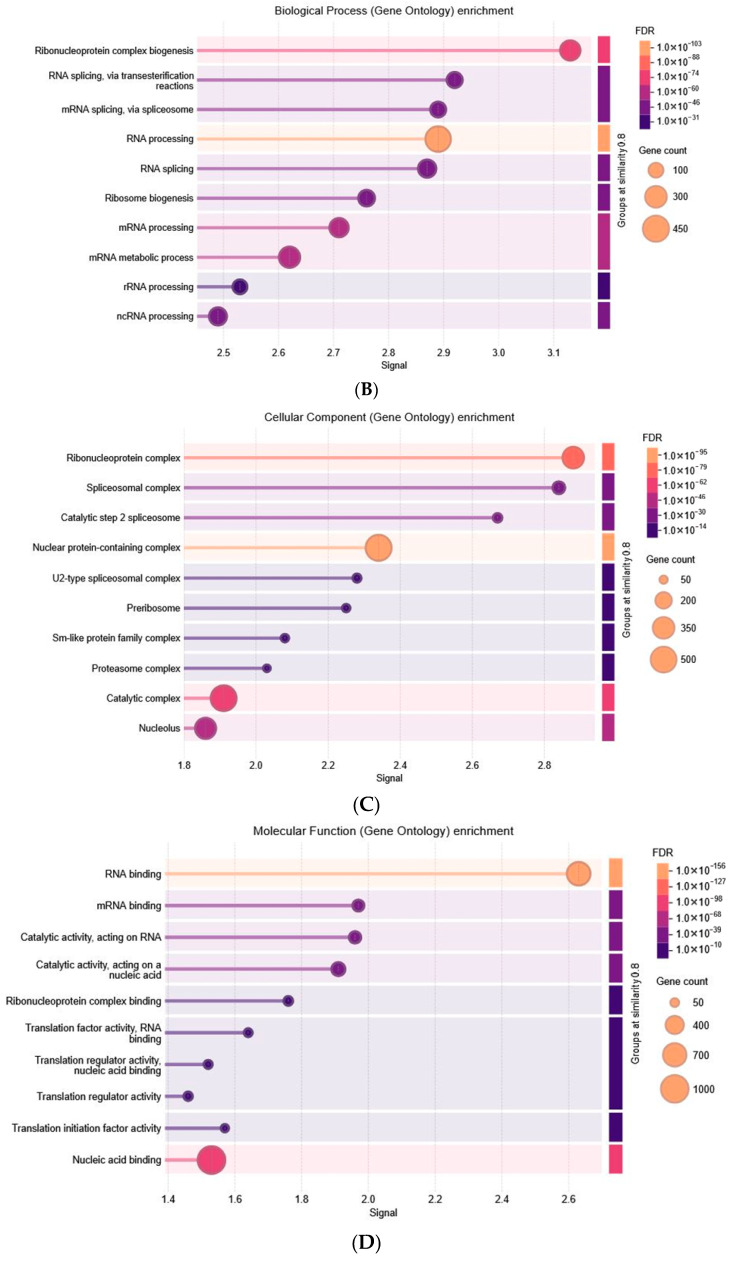
Functional Annotation and Enrichment Analysis of Turquoise Module. (**A**) KEGG, (**B**) GO BP, (**C**) GO CC, (**D**) GO MF.

Enrichment analysis of the turquoise module revealed that its hub genes are central to RNA (Figure 7B) metabolism, ribonucleoprotein assembly, and translational regulation—processes fundamental to both normal cellular function and disease progression. GO biological processes highlighted ribonucleoprotein complex biogenesis, spliceosome-mediated RNA splicing, mRNA processing, ribosome biogenesis, and ncRNA/rRNA processing, underscoring their role in post-transcriptional gene regulation. In terms of molecular function (Figure 7D), these genes were enriched for RNA and mRNA binding, catalytic activity on RNA, ribonucleoprotein complex binding, and translation initiation factor activity, consistent with their in (Figure 7C) involvement in RNA stability, maturation, and protein synthesis. Cellular component enrichment placed them within the spliceosome, preribosome, ribonucleoprotein complexes, nucleolus, and proteasome, pointing to their roles in RNA maturation, ribosome assembly, and protein degradation. KEGG pathway analysis (Figure 7A) further reinforced these findings, showing significant enrichment in the spliceosome, RNA transport, ribosome biogenesis, mRNA surveillance, RNA degradation, and aminoacyl-tRNA biosynthesis pathways—all critical for accurate transcription and translation. Additional enrichment in ubiquitin-mediated proteolysis, proteasome function, cell-cycle control, and the amyotrophic lateral sclerosis (ALS) pathway suggested broader roles in protein quality control and stress responses. Dysregulation of these processes can contribute to cancer development, metabolic imbalance, and neurodegeneration.

### 3.4. Protein–Protein Interaction (PPI) Networks and Hub Genes Analysis

**Figure 8:**
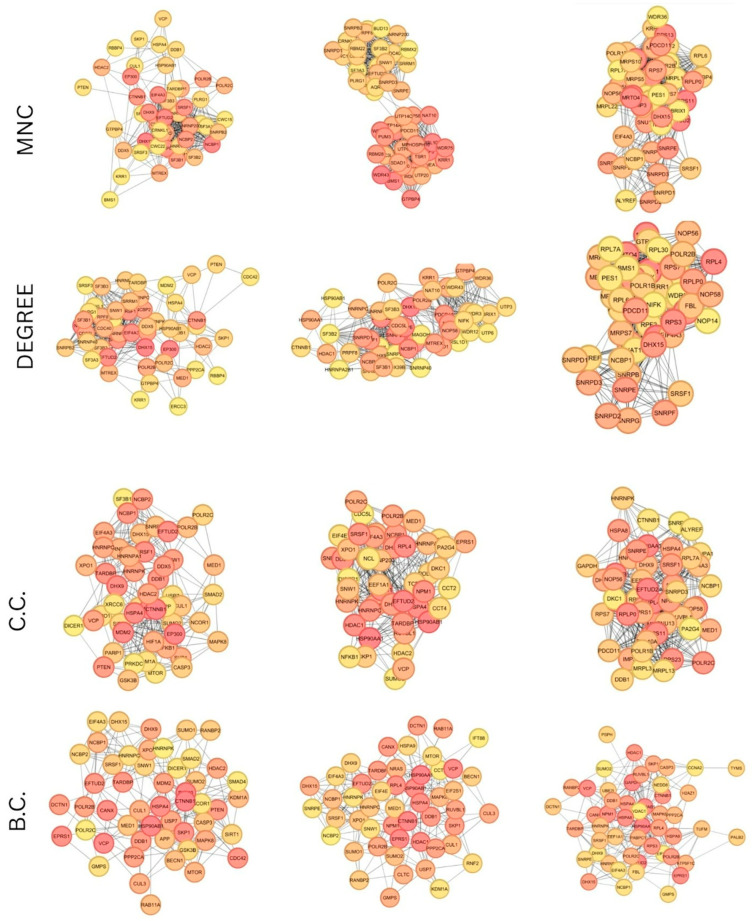
Top 50 hub genes of 3 highly preserved modules (blue, brown, and turquoise) processed using MNC, degree, closeness, and betweenness centrality algorithms of Cytoscape.

**Figure 9:**
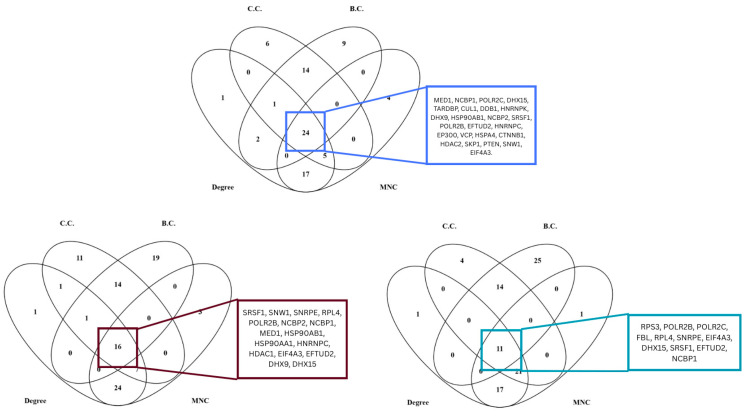
Common hub genes identified by WGCNA and Cytoscape.

Protein–protein interaction (PPI) networks of the genes of every conserved module were generated based on the STRING database by imposing a high-confidence score cutoff (>0.7) to get highly reliable interactions. These derived PPI networks were imported into the software Cytoscape, where the CytoHubba module was used to rank the genes based on various topological measures, i.e., degree, closeness, betweenness centrality, and MNC. The hub genes that were persistently overlapping between these methods of ranking were denoted as the hub nodes that deserve further investigation. As can be observed from Figure 8, the highest interacting score genes were shown by red dots indicating very strong connectivity. These hub genes (Figure 9) would be significantly involved in the underlying biological process and the biological pathways and therefore become the prime focusses of further functional study.

#### 3.4.1. Blue Module Result Analysis

The hub genes identified in the blue module largely converge on transcriptional regulation, RNA processing, and proteostasis—core processes underlying tumorigenesis and metabolic dysfunction. Transcriptional regulators such as MED1, EP300, PTEN, CTNNB1, and HDAC2 shape gene expression, chromatin remodeling, and oncogenic signaling. Meanwhile, factors including NCBP1, NCBP2, POLR2B, POLR2C, DHX15, DHX9, TARDBP, HNRNPK, HNRNPC, SRSF1, EFTUD2, SNW1, and EIF4A3 participate in RNA binding, splicing, and mRNA surveillance. These ensure proper transcript maturation but can also promote oncogenic splicing when dysregulated. Protein homeostasis is maintained by CUL1, SKP1, DDB1, VCP, HSP90AB1, and HSPA4, which regulate ubiquitin-mediated proteolysis, stabilize oncogenic proteins, and support stress responses.

#### 3.4.2. Brown Module

The hub genes of the brown module are enriched in RNA processing, transcription, and protein quality control, highlighting their role in both cancer progression and metabolic dysfunction. Splicing regulators such as SRSF1, SNW1, SNRPE, HNRNPC, EIF4A3, EFTUD2, DHX9, and DHX15 govern pre-mRNA maturation, ribonucleoprotein assembly, and alternative splicing—mechanisms frequently hijacked in colorectal, pancreatic, and renal cancers to favor oncogenic isoforms. Core transcriptional regulators, including POLR2B, NCBP1, NCBP2, and MED1, sustain mRNA synthesis and stability, while RPL4 contributes to ribosome biogenesis and translational control. Protein folding and stress adaptation are supported by HSP90AB1, HSP90AA1, and HDAC1, which stabilize oncogenic proteins and remodel chromatin, while MED1 connects transcriptional coactivation with nuclear receptor signaling.

#### 3.4.3. Turquoise Module

The turquoise module hub genes show a strong convergence on RNA transcription, splicing, and ribosome biogenesis—processes vital for cellular homeostasis but frequently dysregulated in cancer and metabolic disease. Core transcriptional machinery such as POLR2B, POLR2C, and NCBP1 maintain mRNA synthesis and stability, while splicing regulators including SRSF1, EFTUD2, EIF4A3, DHX15, and SNRPE ensure precise pre-mRNA processing via spliceosome assembly. Ribosomal proteins RPS3 and RPL4, together with FBL (fibrillarin), contribute to ribosome biogenesis, rRNA processing, and translation, linking this module to the increased protein synthesis demands of rapidly proliferating cancer cells.

### 3.5. miRNA–Gene Regulatory Interaction of Modules

**Figure 10:**
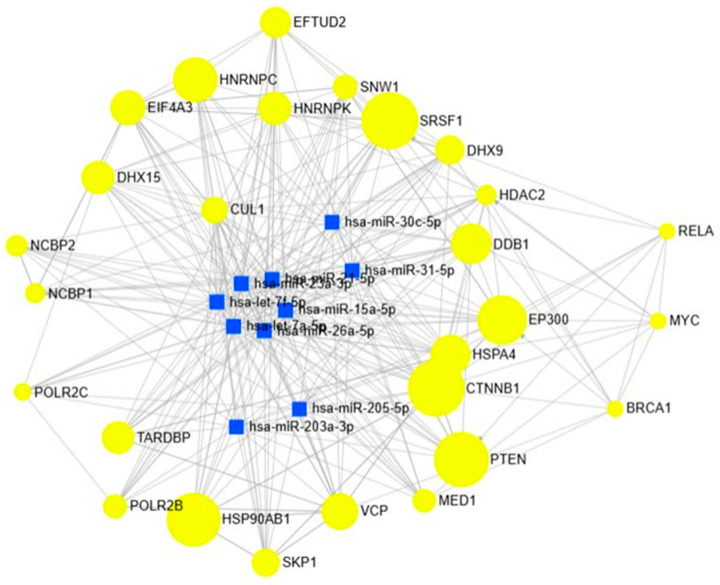
miRNA–Gene Interaction Profiles in the Blue Modules.

Network analysis of the blue module identified 1254 miRNA–gene interactions as shown in Figure 10, with hub miRNAs including hsa-let-7a-5p, hsa-let-7f-5p, hsa-miR-15a-5p, hsa-miR-21-5p, hsa-miR-23a-3p, hsa-miR-26a-5p, hsa-miR-31-5p, hsa-miR-30c-5p, hsa-miR-203a-3p, and hsa-miR-205-5p. Several of these miRNAs are well-documented in the diseases of interest. For instance, miR-21-5p, miR-31-5p, and miR-205-5p are frequently upregulated in colorectal and pancreatic cancers, promoting proliferation, invasion, and resistance to apoptosis. Conversely, let-7 family members act as tumor suppressors but are often downregulated in colorectal cancer and renal cell carcinoma, leading to enhanced oncogene expression. Similarly, miR-15a-5p and miR-23a-3p are implicated in cell cycle control and apoptosis in renal and colorectal cancers, while miR-26a-5p and miR-30c-5p regulate tumor progression and are also linked to diabetic nephropathy and metabolic regulation.

**Figure 11:**
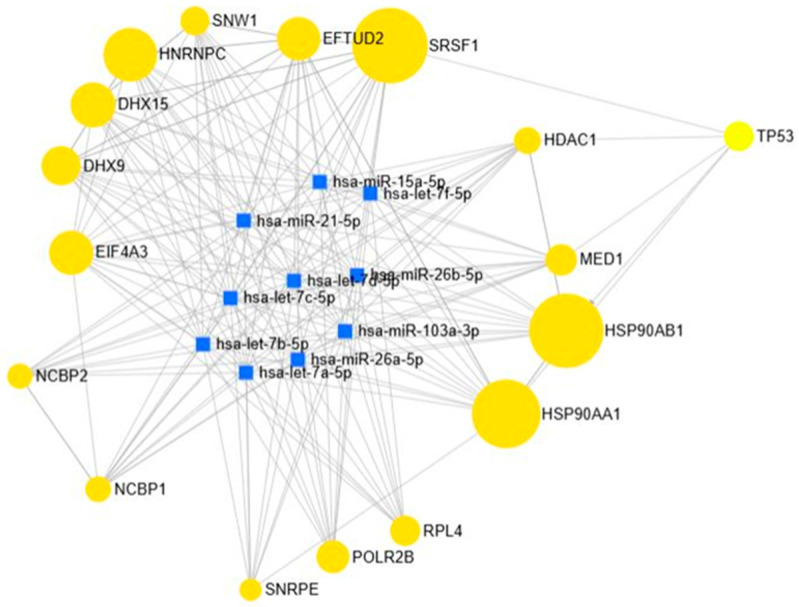
miRNA–Gene Interaction Profiles in the Brown Modules.

The brown module comprised 1039 interactions as shown in Figure 11, with the top 10 hub microRNAs (miRNAs) identified as hsa-let-7a-5p, let-7b-5p, let-7c-5p, let-7d-5p, let-7f-5p, miR-15a-5p, miR-21-5p, miR-26a-5p, miR-26b-5p, and miR-103a-3p. These miRNAs serve as central regulators of oncogenic and metabolic pathways. The let-7 family primarily functions as a tumor suppressor by targeting oncogenes such as RAS and MYC. Frequent downregulation of let-7 family members in colorectal cancer, pancreatic cancer, and renal cell carcinoma facilitates tumor growth and metastasis. miR-15a-5p modulates apoptosis through BCL2 targeting and is commonly dysregulated in colorectal and renal cancers. In contrast, miR-21-5p is a prominent oncomiR, consistently overexpressed in colorectal, pancreatic, and renal cancers, as well as in diabetic kidney disease, where it promotes fibrosis and inflammation. miR-26a-5p and miR-26b-5p demonstrate context-dependent behavior, acting as tumor suppressors in colorectal cancer and contributing to metabolic regulation and kidney injury in diabetes. miR-103a-3p has dual roles, being implicated in insulin resistance and type 2 diabetes, and promoting metastasis in colorectal and pancreatic cancers.

**Figure 12:**
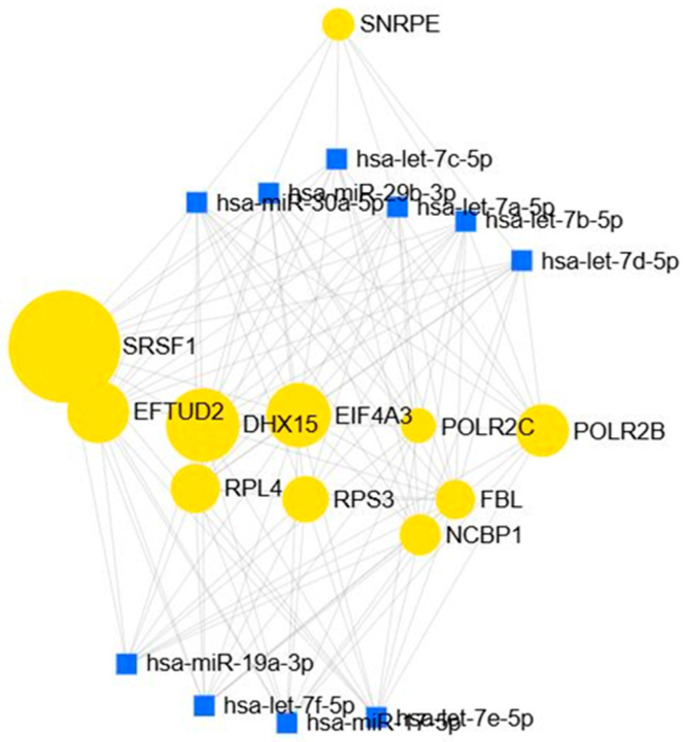
miRNA–Gene Interaction Profiles in the Turquoise Modules.

The turquoise module had 786 interactions shown in Figure 12. The top 10 miRNAs identified—hsa-let-7a/b/c/d/e/f, miR-17-5p, miR-19a-3p, miR-30a-5p, and miR-29b-3p—play important roles in cancer progression and metabolic disease. The let-7 family mainly acts as tumor suppressors by targeting oncogenes like RAS and MYC and controlling the cell cycle. However, these miRNAs are often found at lower levels in colorectal, pancreatic, and renal cancers, which can lead to tumor growth, invasion, and worse outcomes. In contrast, miR-17-5p and miR-19a-3p, which are part of the miR-17-92 cluster, are usually found at higher levels in colorectal and pancreatic cancers, where they promote cell growth, new blood vessel formation, and resistance to cell death. miR-30a-5p generally works as a tumor suppressor by blocking processes like EMT and metastasis in colorectal and renal cancers, and it is also linked to kidney fibrosis in diabetic kidney disease. miR-29b-3p has two roles: it helps prevent extracellular matrix buildup and fibrosis in diabetic nephropathy, but in cancer, changes in its levels can either promote invasion or suppress tumors, depending on the situation.

## 4. Discussion

Gene expression profiling has demonstrated the intricate biological interplay between microinflammation, metabolic disorders, and cancer. Microinflammation, characterized by low-grade, chronic inflammatory activity at the cellular level, initiates a cascade of molecular responses that disrupt homeostasis and promote disease progression. Transcriptomic analyses reveal that this inflammatory state is associated with the overexpression of pro-inflammatory genes and signaling molecules, alongside the under expression or dysregulation of microRNAs that normally act as regulatory brakes on inflammation and cellular proliferation. These gene expression shifts not only contribute to insulin resistance and metabolic dysfunction but also promote a microenvironment conducive for tumorigenesis. The convergence of these pathways underscores a shared molecular architecture among chronic diseases, suggesting that microinflammation may play a central node linking metabolic imbalance to oncogenic transformation.

Colorectal cancer (CRC), pancreatic cancer (PC), and clear cell renal cell carcinoma (ccRCC) are aggressive malignancies that remain major contributors to global cancer mortality. ccRCC, the most prevalent kidney cancer subtype, is characterized by rapid progression, high metastatic potential, and poor prognosis. Similarly, CRC and PC are among the leading causes of cancer-related deaths due to their late detection and strong resistance to therapy. These cancers share common molecular disruptions in RNA processing, transcriptional regulation, and metabolic signaling, reflecting aberrant control of growth, differentiation, and survival pathways. Type 2 diabetes (T2D) and obesity are chronic metabolic diseases marked by impaired insulin signaling, systemic inflammation, and metabolic reprogramming. These alterations not only lead to cardiovascular and endocrine complications but also heighten susceptibility to cancer by promoting oxidative stress, dysregulated glucose metabolism, and enhanced proliferative signaling. The overlap in molecular mechanisms suggests that metabolic stress and oncogenic transformation may be driven by convergent gene networks. Irritable bowel syndrome (IBS) represents a non-malignant yet chronically inflamed state within the gastrointestinal tract, characterized by mucosal immune activation and altered gene expression. Persistent low-grade inflammation in IBS parallels the immune dysregulation seen in obesity, diabetes, and gastrointestinal cancers, further linking inflammatory signaling to metabolic and oncogenic pathways.

### 4.1. Chronic Inflammation as a Central Mechanism

The area surrounding a tumor, called the tumor microenvironment, often becomes inflamed and promotes cancer other inflammation-driven tissue changes that support tumor progression. This microinflammation encourages the growth of new blood vessels (angiogenesis) that feed the tumor, help cancer cells hide from the immune system, and causes damage to DNA through ongoing stress and inflammatory signals [41,42,43]. The tumor microenvironment is characterized not only by inflammatory cytokines but also by profound disruptions in RNA processing, proteostasis, and ribosome biogenesis—the core pathways identified as highly conserved across all disease modules in this study. Dysregulation of spliceosome components such as SRSF1, HNRNPC, and EFTUD2 alters the maturation of transcripts that regulate cell survival, inflammatory signaling, and metabolic Homeostasis [44,45,46]. Likewise, impaired proteostasis, driven by aberrant activity of chaperones (e.g., HSP90AB1) and ubiquitin–proteasome components (CUL1, SKP1), leads to the accumulation of misfolded or damaged proteins, amplifying cellular stress and sustaining pro-tumorigenic signaling [47,48,49]. Enhanced ribosome biogenesis, reflected by upregulation of RPL4, RPS3, and FBL, supports the high translational demand in inflammatory and malignant tissues [50,51]

These core cellular alterations interface directly with inflammatory pathways. Persistent activation of NF-κB and IL-6/STAT3 signaling modifies splicing patterns, increases proteostatic burden, and upregulates ribosome biogenesis, thereby reinforcing the chronic microinflammatory state that promotes tumor initiation and progression [52,53,54]. For example, NF-κB–dependent transcription increases ER stress and proteotoxic load, while STAT3 enhances ribosomal gene transcription and metabolic rewiring. Conversely, defects in RNA and protein quality control further potentiate inflammatory signaling, creating a self- sustaining loop linking microinflammation to oncogenic transformation.

MicroRNAs (miRNAs) play an important role in silencing gene expression activity. One of them, miR-21-5p, is often found at high levels in cancer and can block protective mechanisms, increase inflammation, and make tumors more resistant to therapy [55,56,57]. On the other hand, another group called the let-7 family helps stop cancer growth. When their levels drop, inflammation increases through higher activity of signaling molecules like IL-6, which again encourages tumor development [58,59,60]. In irritable bowel syndrome (IBS), the immune system in the gut lining becomes more active, leading to mild but ongoing inflammation. Studies have found that people with IBS often show higher numbers of immune cells, such as mast cells, as well as small amounts of tissue damage and weakening of the gut’s protective barrier, even without visible disease. These changes resemble what happens in metabolic conditions and cancers, where continuous activation of inflammatory signals creates an environment that supports both metabolic problems and tumor growth [61,62,63,64,65]. When inflammation, cell recycling, and stress-response systems become unregulated, chronic conditions like IBS, obesity, and type 2 diabetes (T2D) can raise the risk of cancers in organs such as the colon, pancreas, and kidneys. These findings also point to possible treatment strategies—such as blocking miR-21-5p, restoring let-7 activity, or targeting enzymes involved in inflammation thereby help reduce long-term inflammation and slow cancer growth.

### 4.2. Metabolic Reprogramming and Cellular Stress

Analysis showed that cancer cells exhibit a marked up-regulation of systems for protein production and maintenance—notably those involved in ribosome biogenesis, mRNA processing/splicing, RNA transport, and ubiquitin-mediated proteolysis [50,66,67,68]. This likely reflects the high demand of tumors for protein synthesis (to support rapid growth) and for proteostasis mechanisms (to manage misfolded or excess proteins under stress) [66,67]. Components of the ribosome and associated RNA-processing machinery make these processes more efficient, while factors such as EP300 may link gene transcription to metabolic control. Through its roles in chromatin remodeling and transcriptional co-activation, EP300 might influence cellular metabolic programs—for example, by modulating glycolytic gene expression or lipid-metabolism genes—thereby helping to meet the metabolic needs of proliferating cells [68,69].

At a finer regulatory layer, certain small non-coding regulators such as microRNAs (miRNAs) may modulate metabolic and proteostatic balance [70,71]. In cancer, some miRNAs reported to be overexpressed enhance energy production and cell growth by targeting metabolic regulators or RNA-binding/splicing factors; conversely, other miRNAs—when downregulated—may impair insulin signaling or promote adipocyte hypertrophy, a mechanism relevant in metabolic diseases like obesity and type 2 diabetes [72,73,74].

Importantly, although our GO/KEGG enrichment did not directly flag classical inflammatory or metabolic signaling pathways (e.g., NF-κB signaling pathway, PI3K/AKT signaling pathway, or JAK/STAT signaling pathway), there is empirical evidence that RNA processing and spliceosome alterations can influence signaling outcomes [75,76,77]. For instance, splicing factors have been shown to regulate alternative splicing of key signaling molecules, including ones in the NF-κB pathway [75,76]. Dysregulation of RNA processing could rewire signaling networks indirectly, e.g., by generating alternative isoforms of signaling proteins with different activity, stability, or localization [76,77].

Thus, rather than asserting a direct enrichment of inflammatory or metabolic signaling pathways, we propose that the “core conserved mechanisms” observed across our modules are the RNA-processing/proteostasis/ribosome biogenesis pathways [75,77]. These may act as a foundation that permits or modulates downstream metabolic reprogramming or inflammatory responses via post-transcriptional regulation (e.g., alternative splicing, miRNA-mediated control, mRNA stability) [75,76,77]. Future work such as a targeted “crosstalk analysis” integrating spliceosome alterations with inflammation/metabolism signaling networks could help to map these indirect links explicitly.

### 4.3. Crosstalk Between Inflammation and Metabolism

Cancer cells take advantage of inflammation to adjust their metabolism and survive under stressful conditions. Inflammatory molecules such as IL-6, TNF-α, and IL-1β send signals that help tumor cells take in more glucose, produce more fats, and change how they use oxygen for energy [75,76,77,78,79]. One key protein involved in this, STAT3, not only affects how genes are turned on but also helps control energy use inside the cell’s power centers, the mitochondria. This allows cancer cells to keep producing energy efficiently and resist damage [78,79,80,81]. Other systems further support fat and protein production, linking inflammation directly to the energy needs of growing tumors [78,82,83]. Together, these processes form the basis of what’s known as the Warburg effect—a pattern where cancer cells prefer to utilize energy pathways, even when oxygen is available [84,85]. These same inflammatory signals also disturb metabolism in conditions like obesity and type 2 diabetes (T2D). Long-term exposure to signaling molecules such as IL-6 and TNF-α places the body’s energy systems in an overactive state, pushing cells to rely more on sugar breakdown and fat production while reducing their ability to use oxygen efficiently. This leads to insulin resistance, enlargement of fat cells, and higher levels of stress within the body [73,82,86,87]. Such “cancer-like” energy changes have also been found in long-term inflammatory and neurological disorders [88,89].

The gut microbiota is now studied to play a crucial role in this connection between inflammation and metabolism. When the balance of these microbes is disrupted, it reduces the production of valuable compounds like short-chain fatty acids (SCFAs), especially butyrate which normally protects the gut lining and helps control inflammation, while harmful substances such as lipopolysaccharides (LPS) from certain bacteria can leak into the bloodstream and trigger inflammation and insulin resistance [90,91,92,93,94]. This imbalance—less butyrate and more LPS—creates a dangerous cycle of gut barrier damage, immune activation, and metabolic stress [95,96,97,98]. These findings show that inflammation and metabolism are highly interconnected. Inflammatory signals lead cells into energy states like those seen in tumors, while changes in gut microbes render the inflammation continuous. Treatments that break this cycle—such as reducing the activity of key inflammatory proteins, restoring healthy SCFA levels, or blocking harmful bacterial signals—could help bring metabolism back into balance in conditions like IBS, obesity, and T2D, while also lowering the risk of cancer.

### 4.4. Clinical and Translational Implications

Key molecules identified in this study could serve as useful indicators for early detection and treatment of diseases that link metabolism and cancer. For instance, the protein SRSF1 is often found at high levels in colorectal and other solid tumors, where it helps cancer cells grow and spread. Because of this, SRSF1 could be a strong candidate for use in early diagnosis and predicting how a tumor will behave [90,91,92,93]. In contrast, PTEN, a protective protein that helps control cell growth, is frequently lost in many cancers, and its absence is associated with poor treatment outcomes. Doctors already use PTEN status to guide treatment in prostate and kidney cancers, showing its real-world clinical importance [99,100]. Another molecule, POLR2B, which helps control how genetic information is copied and used, has been linked to worse outcomes in kidney and other cancers, further emphasizing the role of gene regulation in tumor aggressiveness [101,102,103].

Beyond traditional genes, small molecules called microRNAs (miRNAs) show great promise as easy-to-detect biomarkers. They can be found in blood or stool samples, making testing less invasive. For example, miR-21-5p is one of the most studied cancer-related miRNAs and is useful for identifying both colorectal cancer and diabetic kidney disease. Meanwhile, low levels of the let-7 family are linked to a loss of protective, tumor-suppressing activity, and changes in miR-29, especially miR-29b-3p, are connected to tissue scarring and increased cancer risk. When combined in diagnostic panels, these miRNAs significantly improve the accuracy of tests for colorectal cancer, particularly in noninvasive screenings [27,104,105,106,107,108]. Table 2 summarizes the key hub genes and regulatory microRNAs identified in this study, highlighting their associated biological pathways, related diseases, and functional roles in linking inflammation, metabolism, and cancer progression.

From a treatment perspective, targeting inflammation and metabolism together offers promising strategies. Anti-inflammatory drugs that block signals like NF-κB or key cytokines can reduce the constant inflammation that supports cancer growth and disrupts normal metabolism. For instance, bortezomib, a drug that interferes with protein breakdown, can suppress tumor survival signals, while cytokine blockers such as canakinumab (which targets IL-1β) and tocilizumab (which targets IL-6) have shown the potential to lower cancer risk and improve response to therapy [109,110,111,112,113,114,115,116].

Medications that affect metabolism also show benefits. Metformin, a common diabetes drug, has demonstrated anticancer effects by helping regulate energy use in cells, while everolimus, another metabolic regulator, is already used to treat kidney cancers [117,118,119,120]. New treatments are also emerging using miRNA-based therapy, which can either block harmful miRNAs or restore beneficial ones. For example, drugs that silence miR-21 or replace lost let-7 activity are being tested, and early trials—like those for lademirsen (an anti–miR-21 drug)—show encouraging results. Advanced delivery systems using nanoparticles are helping make these therapies more precise and effective [121,122,123,124]. These findings highlight how the same molecules can serve both as biomarkers for diagnosis and as targets for treatment. By understanding how inflammation, metabolism, and gene regulation interact, researchers can design new tests and therapies that address the shared biological mechanisms driving both metabolic diseases and cancer.

Although these approaches show promise, repurposing drugs across different diseases warrants caution due to tissue-specific expression of hub genes, varying pharmacodynamics, and potential off-target effects. Thus, the translational implications of our in silico findings should be considered as a foundation for hypothesis-driven experimental and clinical validation rather than immediate clinical application.

## 5. Conclusions

This study shows that irritable bowel syndrome (IBS), obesity, type 2 diabetes (T2D), colorectal cancer (CRC), renal cell carcinoma (RCC), and pancreatic cancer are underpinned by highly conserved molecular networks. At the core of these networks are hub genes and regulatory miRNAs that connect chronic inflammation, metabolic reprogramming, and cellular stress responses. Using weighted gene co-expression network analysis (WGCNA), we identified three major preserved modules (blue, brown, turquoise) enriched in pathways related to RNA processing, spliceosome function, ribosome biogenesis, and proteostasis regulation. These modules bring together transcriptional regulators such as MED1, EP300, and PTEN, ribosomal proteins including RPL4 and RPS3, and splicing factors like SRSF1 and EFTUD2, alongside miRNAs such as miR-21-5p, the let-7 family, miR-29b-3p, and miR-103a-3p, highlighting central axes of disease crosstalk. Functionally, chronic low-grade inflammation emerges as a key driver. The gut microbiota further amplifies these effects by shaping inflammatory signaling and altering metabolite availability.

In conclusion, our gene expression analysis underscores the profound interconnectivity between microinflammation, cancer, and metabolic diseases. The upregulation of genes encoding signaling molecules reveals a complex network of alternative and sustained pathways that drive various pathological processes, including cellular proliferation, immune modulation, and metabolic dysregulation. Notably, the concurrent data on the downregulation of specific microRNAs further exacerbates these effects by removing critical regulatory checkpoints in the said pathways. This dual modulation contributes to a state of alternative metabolic reprogramming, which has emerged as a pivotal mechanism in the initiation and progression of both metabolic disorders and various malignancies. Our findings provide insights into the gene expression dynamics underlying these conditions and highlight microinflammation as a shared biological mechanism that connects multiple chronic diseases. These insights open avenues for targeted therapeutic strategies aimed at intercepting shared inflammatory and metabolic pathways across disease spectra.

## Figures and Tables

**Table 1:** Information of the GEO datasets used in the study.

Accession No.	GSE146853	GSE164416	GSE152991	GSE213324	GSE50760	GSE211398
Condition	Irritable Bowel Syndrome (IBS)	Type 2 Diabetes	Obesity	Renal Cell Carcinoma (RCC)	Colorectal Cancer	Pancreatic Cancer
Type	Expression profiling by high-throughput sequencing					
Source	Colon Tissue	Pancreatic Tissue	Subcutaneous adipose tissue	Kidney Tissue	Colon Tissue and Liver Metastasis	Pancreatic Tissue
Samples	46 cases	37 cases	34 cases	21 tumors	36 tumors	16 tumors

**Table 2:** Summary of Key Genes with Their Associated Pathways, Diseases, and Biological Roles.

Gene/miRNA	Associated Pathway/Process	Associated Disease(s)	Brief Functional Role	Therapeutic Approach/Drug	Mechanism of Action	Key Clinical Outcome
SRSF1	RNA splicing, mRNA processing	CRC, PC, ccRCC	Splicing factor that promotes oncogenic alternative splicing events, enhancing cell proliferation, invasion, and tumor progression.	Antisense oligonucleotides	Modulates aberrant splicing	Reduced oncogenic splicing events in vitro
PTEN	PI3K/AKT signaling, NF-κB regulation	CRC, PC, ccRCC, obesity, T2D	Tumor suppressor that inhibits PI3K/AKT; its loss activates NF-κB, promotes inflammation, and supports tumor growth and insulin resistance.	Metformin	Activates AMPK, restores metabolic regulation	Improved metabolic balance in CRC
CTNNB1	Wnt/β-catenin signaling, NF-κB crosstalk	CRC, PC, ccRCC	Promotes cell proliferation, immune evasion, and metastasis through interaction with inflammatory signaling.	Tankyrase inhibitors	Blocks Wnt/β-catenin pathway	Reduced tumor growth and metastasis in vitro
HDAC2	Epigenetic regulation, chromatin remodeling	CRC, PC, ccRCC	Co-activator linking transcriptional activity with metabolism; promotes glycolysis and lipogenesis in cancer cells.	HDAC inhibitors	Inhibits HDAC, restores normal transcription	Reduced tumor proliferation in multiple cancers
POLR2B	Transcriptional regulation, RNA polymerase II activity	ccRCC, other solid tumors	Subunit of RNA polymerase II; its dysregulation enhances transcriptional output and tumor aggressiveness.	CDK7 inhibitors	Suppresses transcriptional hyperactivation	Reduced tumor cell proliferation in vitro
RPL4/RPS3/FBL	Ribosome biogenesis, translation	CRC, PC, ccRCC, obesity, T2D	Support increased protein synthesis and stress adaptation in malignant and metabolically active cells.	None reported	—	—
miR-21-5p	NF-κB/PI3K/AKT signaling	CRC, T2D, ccRCC	OncomiR that suppresses PTEN, amplifies inflammatory signaling, and promotes tumor growth and therapy resistance.	Lademirsen (anti-miR-21)	miRNA inhibition	Reduced serum miR-21 levels
let-7 family	IL-6/RAS/MYC regulation	CRC, PC, ccRCC, obesity	Tumor-suppressive miRNA family that inhibits oncogenes; its loss increases IL-6 signaling, inflammation, and proliferation	let-7 mimics	Restores tumor suppressor activity	Enhanced apoptosis, reduced proliferation
miR-29 family	Fibrosis/ECM remodeling/energy balance	CRC, obesity, T2D	Regulates extracellular matrix turnover and fibrosis; its depletion leads to tissue remodeling and metabolic dysfunction.	miR-29 mimics	Restores miRNA function	Reduced fibrosis and ECM deposition
miR-103/miR-107	Insulin signaling, glucose metabolism	Obesity, T2D	Impair insulin receptor signaling and promote insulin resistance; also linked to metabolic stress pathways.	Antisense oligonucleotides	miRNA inhibition	Improved insulin sensitivity in vitro
miR-17-92 cluster	c-Myc/Wnt/β-catenin signaling	CRC, PC	Amplified in cancers; enhances glycolysis and anabolic growth through oncogenic transcriptional networks	AntagomiRs targeting miR-17-92	miRNA inhibition	Reduced tumor proliferation in vitro

## Data Availability

The transcriptomic datasets analyzed in this study are publicly available from the Gene Expression Omnibus (GEO) database (https://www.ncbi.nlm.nih.gov/geo/, accessed on 3 June 2025). The datasets include irritable bowel syndrome (GSE146853), Type 2 Diabetes (GSE164416), Obesity (GSE152991), Renal Cell Carcinoma (GSE213324), Colorectal Cancer (GSE50760), and Pancreatic Cancer (GSE211398).

## Abbreviations

The following abbreviations are used in this manuscript:

T2D	Type 2 diabetes
IBS	Irritable bowel syndrome
CRC	Colorectal cancer
RCC	Renal cell carcinoma
PC	Pancreatic cancer
CCRCC	Clear cell renal cell carcinoma
WGCNA	Weighted Gene Co-Expression Network Analysis
